# Clinical condition and comorbidity as determinants for blood culture positivity in patients with skin and soft-tissue infections

**DOI:** 10.1007/s10096-017-3001-0

**Published:** 2017-06-07

**Authors:** F. V. van Daalen, M. C. Kallen, C. M. A. van den Bosch, M. E. J. L. Hulscher, S. E. Geerlings, J. M. Prins

**Affiliations:** 1 Department of Internal Medicine, Division of Infectious Diseases, Academic Medical Centre, University of Amsterdam, Room F4-132, Meibergdreef 9, 1105 AZ Amsterdam, the Netherlands; 2 Department of Scientific Institute for Quality of Healthcare (IQ healthcare), Radboud University Medical Center, Nijmegen, the Netherlands

## Abstract

The utility of performing blood cultures in patients with a suspected skin infection is debated. We investigated the association between blood culture positivity rates and patients’ clinical condition, including acute disease severity and comorbidity. We performed a retrospective study, including patients with cellulitis and wound infection who had been enrolled in three Dutch multicenter studies between 2011 and 2015. Patients’ acute clinical condition was assessed using the Modified Early Warning Score (MEWS; severe: MEWS ≥2) and comorbidity with the Charlson Comorbidity Index (CCI; severe: CCI ≥2). A total of 334 patients with a suspected skin infection were included. Blood cultures were performed in 175 patients (52%), 28 of whom (16%) had a positive blood culture. Data on the clinical condition were collected in 275 patients. Blood cultures were performed in 76% of the patients with a severe acute condition, compared with 48% with a non-severe acute condition (OR 3.5; 95% confidence interval: 2.0–6.2; *p* < 0.001). Blood cultures were positive in 18% and 12% respectively (OR 1.7 (0.7–4.1); *p* = 0.3). Blood cultures were performed in 53% of patients with severe comorbidity, compared with 61% without severe comorbidity (OR 0.7; 0.4–1.2; *p* = 0.2). Blood cultures were positive in 25% and 10% respectively (OR = 3.1; 1.2–7.5; *p* = 0.02). The blood culture positivity rate among hospitalized patients diagnosed with skin infections was higher than the rates reported by the Infectious Diseases Society of America guidelines, particularly in patients with severe comorbidity. Therefore, the recommendations concerning blood culture performance in patients with a skin infection should be reconsidered.

## Introduction

Acute bacterial skin and soft-tissue infections (SSTI) are among the most common indications for antibiotic use in hospitalized adults [1]. Cellulitis involves the deep dermis and subcutaneous tissues, whereas erysipelas is limited to superficial dermal structures with well-defined borders, although it is presently considered a manifestation of cellulitis [2–5]. Most of these infections arise from beta-hemolytic streptococci. *Staphylococcus aureus* can cause cellulitis, typically in the presence of an open wound or previous penetrating trauma. Several other organisms can also cause cellulitis, but usually only in special circumstances [2]. Wound infections are defined by the presence of a pre-existing skin lesions at the time of skin infection onset, such as a chronic ulcer or a trauma-related skin lesion, and can vary from superficial skin infection to deeper infection with involvement of tissues under the skin [6, 7].

The current Infectious Diseases Society of America (IDSA) guidelines do not recommend routine performance of blood cultures in patients with cellulitis and erysipelas [2]. Previous studies reported low positivity rates (4–13%) and marginal consequences for treatment, as standard empirical therapy covers most causative pathogens, including beta-hemolytic streptococci [2, 3, 8–11].

Although *S. aureus* and gram-negative bacteria are less frequently noted to cause SSTI, they require different treatment [2, 9]. As these pathogens are more frequently found in specific patient groups, IDSA guidelines recommend performing blood cultures only in patients with malignancy, chemotherapy, neutropenia, severe cell-mediated immunodeficiency, immersion injuries, and animal bites [2]. These recommendations, however, are based on little evidence and do not take the patients’ acute clinical condition into account.

The aim of our study was to investigate the association between blood culture positivity rates and the patient’s clinical condition, including acute disease severity and comorbidity.

## Materials and methods

### Study setting and population

We performed a retrospective study in the databases of two multicenter observational studies (RIANT and IMPACT) and one multicenter intervention study (AB checklist), performed between 2011 and 2015 in 40 university and non-university hospitals in the Netherlands (http://www.trialregister.nl; NTR 5933) [12, 13]. The RIANT study developed and validated a set of generic quality indicators to define and measure the appropriateness of antibiotic use in the treatment of bacterial infections in hospitalized adult patients. The IMPACT study compares different methods, including a point prevalence survey using these validated quality indicators, to measure the appropriateness of antibacterial use in hospitalized adult patients. The AB checklist study implemented an antibiotic checklist based on these validated quality indicators and evaluated the effect of checklist use on the length of hospital stay and appropriate antibiotic use. Inclusion criteria for all three studies were: adult patients (≥18 years old) admitted to an acute care department with a suspected bacterial infection and treated with antibiotics (ATC group J01) for at least 24 hours. In all three studies, patients were excluded if antibiotics were used as prophylaxis, or when antibiotic treatment was started on the ICU department or in another hospital. All patients were randomly selected. For the present study, we identified the patients with cellulitis in these databases, including those with a clinical diagnosis of erysipelas; we also included patients with a wound infection.

### Assessments

Data were collected from electronic medical records and medication charts. In all three studies, data were recorded on patient characteristics, including sex, age and diagnosis, and whether blood culture had been performed, including the number of cultures and their results.

In two studies, RIANT and AB checklist, additional data were recorded on the acute clinical condition and comorbidity [12, 13]. Patients’ acute clinical condition was assessed at the start of treatment using the Modified Early Warning Score (MEWS) [14]. A severe acute condition was defined by MEWS ≥2, non-severe acute condition by MEWS <2. Comorbidity was assessed using the Charlson Comorbidity Index (CCI) [15] and severe comorbidity was defined by CCI ≥2.

### Analysis

Numbers of blood cultures taken and numbers of positive cultures, including type of pathogen, were combined for all three studies and expressed in absolute numbers and percentages. We compared the proportions of positive blood cultures between patients diagnosed with cellulitis, those with a clinical diagnosis of erysipelas, and those with a wound infection. In the evaluation of the association between blood culture positivity rates and patients’ clinical condition we only included patients of the RIANT and AB checklist studies [12, 13]. We compared the proportions of patients with a blood culture taken and positivity rates between patients with a severe and those with a non-severe acute clinical condition, and between patients with and without severe comorbidity. Additionally, we performed a sub-analysis in which we compared patients with or without diabetes mellitus. We used Chi-squared tests to compare proportions. As age and comorbidity are often correlated, we performed a multivariate logistic regression analysis to investigate the association between the positivity rate of blood cultures and acute clinical condition, comorbidity and age of the patient. *P* < 0.05 was considered statistically significant. Analyses were carried out using IBM SPSS Statistics, version 23.0.

## Results

A total of 334 patients with a suspected SSTI were included in our study, of whom 258 (77%) were diagnosed with cellulitis and 76 (23%) with a wound infection. Blood cultures were performed in 175 out of 334 patients (52%): in 153 out of 258 patients (59%) with cellulitis and in 22 out of 76 patients (29%) with a wound infection. Twenty-eight out of 175 patients (16%) had a positive blood culture: 23 out of 153 patients (15%) with cellulitis and 5 out of 22 with a wound infection (23%). One hundred and nine of the patients with cellulitis (42%) had a clinical diagnosis of erysipelas. Blood cultures were performed in 70 out of 109 patients (64%) with erysipelas and 8 out of 70 (11%) had a positive blood culture. Table 1 presents the baseline characteristics of all patients, of patients in whom blood cultures were performed, and of patients with positive blood cultures.

**Table 1:** Baseline characteristics of patients with a suspected skin and soft-tissue infection (SSTI)

Characteristics^a^	All patients	Patients in whom blood cultures were performed	Patients with positive blood cultures
	*n* = 334	*n* = 175	*n* = 28
Sex, male	181 (55)	96 (55)	15 (54)
Age, mean (SD)	66.0 (17)	65.1 (16)	69.7(16)
Hospital			
University	42 (13)	17 (10)	3 (11)
Non-university	292 (87)	158 (90)	25 (89)
Diagnosis			
Cellulitis	258 (77)	153 (87)	23 (82)
Erysipelas subgroup	109 (33)	70 (40)	8 (29)
Wound infection	76 (23)	22 (13)	5 (18)
Antibiotics			
Started intravenously (IV)	312 (93)	172 (98)	28 (100)
Started orally (O)	22 (7)	3 (2)	0 (0)
Total	334 (100)	175 (100)	28 (100)

^a^Numbers are *n* (%), unless otherwise indicated

No significant difference was found in positivity rates between the patients with a diagnosis of cellulitis not classified as erysipelas (*n* = 83) and those with a clinical diagnosis of erysipelas (*n* = 70; OR 0.6; 95% confidence interval: 0.2–1.5; *p* = 0.3), or between patients with cellulitis (*n* = 153) and those with a wound infection (OR 0.6; 0.2–1.8; *p* = 0.4).

Twenty-nine pathogens were identified. Most cultured species were *Streptococcus* species (in 12 cultures), Gram-negative bacteria (in 8 cultures), and *Staphylococcus aureus* (in 7 cultures). See Table 2 for details per diagnosis.

**Table 2:** Microbiology results of patients with a suspected SSTI and a positive blood culture

Pathogen	Total number of pathogens^a^	Number of pathogens per diagnosis^a^		
		Cellulitis		Wound infection
			Erysipelas subgroup	
Negative culture results				
No pathogen	140 (96)	125	59	15
Contamination	6 (4)	5	3	1
Total	146 (100)	130	62	16
Positive culture results				
*Staphylococcus* species				
*S. aureus*	7 (24)	4		3
*S. capitis*	1 (3)	1	1	
*Streptococcus* species				
*S. pneumoniae*	1 (3)	1		
*S. pyogenes*	3 (10)	3	1	
*S. dysgalactiae*	2 (7)	2	1	
*S. salivarius*	1 (3)			1
Group A	1 (3)	1		
Group B	2 (7)	2		
Group C	1 (3)	1		
Group G	1 (3)	1	1	
Enterococcus species				
*E. faecium*	1 (3)	1		
Gram-negative bacteria				
*E. coli*	1 (3)			1
*K. oxytoca*	2 (7)	2	1	
*P. aeruginosa*	3 (10)	3	1	
*Pseudomonas* species	1 (3)	1	1	
*H. influenza*	1 (3)	1	1	
Total	29^b^ (100)	24	8	5

^a^Numbers are *n* (%), unless otherwise indicated

^b^In one patient two pathogens were identified

Data on acute clinical condition and comorbidity were recorded in 275 patients, of whom 214 (78%) were diagnosed with cellulitis and 61 (22%) with a wound infection. Eighty-five out of 275 (31%) had a severe acute clinical condition, 102 out of 275 (37%) had severe comorbidity. Table 3 presents the baseline characteristics of these patients. Blood cultures were performed in 76% of the patients with a severe acute clinical condition, compared with 48% of patients with a non-severe condition (OR 3.5; 1.9–6.2; *p* < 0.001). Blood cultures were positive in 18% and 12% respectively (OR 1.7; 0.7–4.1; *p* = 0.3). In 53% of the patients with a severe comorbidity blood cultures were performed, compared with 61% of patients without a severe comorbidity (OR =0.7; 0.4–1.2; *p* = 0.2). Blood cultures were positive in 25% and 10% respectively (OR = 3.1; 1.2–7.5; *p* = 0.02; Table 4).

**Table 3:** Baseline characteristics of 275 patients in the clinical condition analyses

Characteristics^a^	Patients included in the acute clinical condition analysis		Patients included in the comorbidity analysis	
	MEWS <2	MEWS ≥2	CCI <2	CCI ≥2
	*n* = 190	*n* = 85	*n* = 173	*n* = 102
Sex, male	112 (59)	36 (42)	102 (59)	46 (45)
Age, mean (SD)	66.1 (17)	66.2 (17)	61.6 (17)	73.8 (14)
Hospital				
University	19 (10)	10 (12)	15 (9)	14 (14)
Non-university	171 (90)	75 (88)	158 (91)	88 (86)
Antibiotic at start of treatment				
Flucloxacillin	98 (52)	40 (47)	99 (57)	39 (38)
Amoxicillin-clavulanic acid	39 (21)	18 (21)	33 (19)	24 (23)
Clindamycin	17 (9)	3 (4)	10 (6)	10 (10)
Penicillin	10 (5)	8 (9)	12 (7)	6 (6)
Cefuroxime	6 (3)	3 (4)	6 (3)	3 (3)
Ceftriaxone	2 (1)	2 (2)	1 (1)	3 (3)
Other	18 (9)	11 (13)	12 (7)	17 (17)
IV / oral				
Started intravenously (IV)	179 (94)	79 (93)	169 (98)	89 (87)
Started orally (O)	11 (6)	6 (7)	4 (2)	13 (13)
Length of stay, days, geometric mean (95%CI)	7.0 (6.2–7.9)	8.3 (6.8–10.2)	5.9 (5.2–6.6)	10.9 (9.1–13.0)
In-hospital mortality rate	6 (3)	2 (2)	2 (1)	6 (6)

*MEWS* Modified Early Warning Score, *CCI* Charlson Comorbidity Index

^a^Numbers are *n* (%), unless otherwise indicated

**Table 4:** The MEWS and CCI of patients in the clinical condition analysis

	Total number of patients	Number of patients in whom blood cultures were performed^a^	Number of patients with positive blood cultures^a^
MEWS			
Non-severe (<2)	190	92 (48)	11 (12)
Severe (≥2)	85	65 (76)	12 (18)
Total	275	157 (57)	23 (15)
Odds ratio, 95% confidence interval, *p* value		3.5, 2.0–6.2, *p* < 0.001^b^	1.7, 0.7–4.1, *p* = 0.3^b^
CCI			
Non-severe (<2)	173	104 (60)	10 (6)
Severe (≥2)	102	53 (52)	13 (13)
Total	275	157 (57)	23 (15)
Odds ratio, 95% confidence interval, *p* value		0.7, 0.4–1.2, *p* = 0.2^c^	3.1, 1.2–7.5, *p* = 0.02^c^

^a^Numbers are *n* (%), unless otherwise indicated

^b^Nonsevere MEWS is the reference group

^c^Nonsevere CCI is the reference group

Of the 275 patients included in the comorbidity analysis, 73 were diagnosed with diabetes mellitus. Blood cultures were performed in 44 of these patients. Blood cultures were positive in 9 out of 44 of the patients with diabetes (20%), compared with 14 out of 113 (13%) of the patients without diabetes (OR 1.8; 0.7–4.6; *p* = 0.2).

The mean age of patients with severe comorbidity was higher than the mean age of patients without severe comorbidity (Table 4). A higher age was significantly associated with positive blood cultures (OR = 1.03; 1.0–1.06; *p* = 0.07). In the multivariate regression analysis, the severity of comorbidity was a stronger predictor of positive blood cultures than age (CCI: OR 2.3; 0.9–6.2; *p* = 0.09, compared with age: OR 1.0; 1.0–1.1; *p* = 0.3).

## Discussion

In this study, we found that blood cultures were taken in 52% of patients hospitalized with cellulitis or wound infections, with a positivity rate of 16%. Significantly more blood cultures were performed in patients with a severe compared with patients with a non-severe acute clinical condition, but positivity rates of blood cultures were significantly higher in patients with severe comorbidity.

The positivity rate in our study was higher than in previous studies, where blood cultures were found to be positive in 4–9% of patients with cellulitis or erysipelas [2, 8, 9, 11]. In one study, higher positivity rates were found for purulent compared with nonpurulent infections [7]. Definitions and inclusion criteria differed between studies, which may explain the range in positivity rates.

Our study supports previous findings stating that *Streptococci species* are the most frequently cultured species in patients with cellulitis and erysipelas, followed by *S. aureus* and Gram-negative bacteria [9]. *S. aureus* was cultured in 3 of the 5 patients with a wound infection and a positive blood culture, in line with the literature describing that this pathogen is typically found in patients with an open wound or previous penetrating trauma [2]. Remarkably, in 4 out of 8 patients with a clinical diagnosis of erysipelas and a positive culture, Gram-negative bacteria were cultured.

Clinicians were more likely to perform blood cultures in patients with a severe acute clinical condition, defined by a MEWS ≥2. A recent Swedish study described a similar association, showing that blood cultures were more frequently performed in patients who fulfilled the severe inflammatory response syndrome (SIRS) criteria [8]. However, they did not compare the positivity rates of blood cultures between patients who did or did not fulfill the SIRS criteria. An American retrospective chart review did look at positivity rates and acute clinical condition: they compared—among others—positivity rates of blood cultures in patients with cellulitis with or without fever [11]. Unexpectedly, patients without fever had positive blood cultures significantly more frequently than patients with fever, which supports our findings that a severe acute clinical condition is not associated with a higher positivity rate.

In our study clinicians were not more likely to perform blood cultures in patients with severe comorbidity, defined by a CCI ≥2, but the positivity rate was significantly higher in these patients. An association between comorbidity and positivity rates has been described before, especially for malignancy, immunodeficiency or diabetes mellitus [2, 10]. Diabetes mellitus is a comorbidity with a high prevalence and often contributes to the total CCI score. A relationship between diabetes mellitus and positive blood cultures has been described [16]. In our study, the positivity rate for patients with diabetes was also higher than in patients without diabetes, but this difference was not statistically significant. As age is the most important risk factor for the presence of comorbidities, an association between the patient’s age and the positivity rate of blood cultures is plausible. The multivariate regression analysis illustrated, however, that comorbidity is a better predictor of blood culture positivity.

Our study has several strengths. We provide recent data on blood culture positivity rates in patients hospitalized with an SSTI, which is necessary as the IDSA guidelines still report positivity rates ≤5% based on a study from 1999 [10]. Our study is large and representative, as we included patients from nearly half of all Dutch hospitals, including university, teaching, and general hospitals.

Our study has limitations. As it was based on retrospective chart reviews, it was the clinicians’ decision as regards which patients’ blood cultures were performed. Our data clearly show that clinicians are more inclined to perform blood cultures in severely ill patients. However, we also showed that positivity rates were better predicted by comorbidity. Furthermore, because of this retrospective setup, we selected patients based on the diagnosis documented in the patient files and we were dependent on the documentation of health care providers.

In conclusion, the blood culture positivity rate among hospitalized patients diagnosed with SSTIs was higher than the rates reported by the IDSA guidelines, particularly in patients with severe comorbidity. Therefore, the recommendations concerning blood culture performance in patients with an SSTI should be reconsidered.
